# The concept of "compartment allergy": prilocaine injected into different skin layers

**DOI:** 10.1186/1710-1492-7-7

**Published:** 2011-04-20

**Authors:** Marion Wobser, Zeno Gaigl, Axel Trautmann

**Affiliations:** 1 Department of Dermatology, Venereology, and Allergology, University of Wuerzburg, Germany

## Abstract

We herein present a patient with delayed-type allergic hypersensitivity against prilocaine leading to spreading eczematous dermatitis after subcutaneous injections for local anesthesia with prilocaine. Prilocaine allergy was proven by positive skin testing and subcutaneous provocation, whereas the evaluation of other local anesthetics - among them lidocaine, articaine and mepivacaine - did not exhibit any evidence for cross-reactivity.

Interestingly, our patient repeatedly tolerated strictly *deep *subcutaneous injection of prilocaine in provocation testing while patch and *superficial *subcutaneous application mounted strong allergic responses. We hypothesize, that lower DC density in deeper cutaneous compartments and/or different DC subsets exhibiting distinct functional immunomodulatory properties in the various layers of the skin may confer to the observed absence of clinical reactivity against prilocaine after *deep *subcutaneous injection.

The term compartment allergy indicates that the route of allergen administration together with the targeted immunologic environment orchestrates on the immunologic outcome: overt T-cell mediated allergy or clinical tolerance.

## Background

Local anesthetics (LA) are extensively used drugs with a safe application profile and only rare objective side effects. Most reported adverse reactions can be attributed to inherent pharmacological and toxic effects of the LA - especially after applying high doses or in case of accidental intravasal injection - as well as psychovegetative disturbance merely due to the painful procedure. Immune-mediated reactions comprise less than 1% of all adverse LA reactions whereby true IgE-mediated allergic reactions to LA are very rare - if they occur at all [1,2].

Delayed-type hypersensitivity reactions to LA are thought to occur more commonly than immediate reactions. The diverse antigenic determinants of the different LA substance groups (esters, amides) and their metabolites as well as the exact immunologic mechanisms involved in delayed-type reactions to LA are partially elaborated. Therefore, putative cross-reactivity between the different substance classes can be predicted merely from the LA structure. Allergic reactions are most often caused by ester compounds of LA putatively because of its metabolite paraaminobenzoeacid (PABA) as the relevant antigenic structure [3]. Delayed-type hypersensitivity against LA may be acquired by different exposure routes. While epicutaneous application in ointments may lead to sensitization via epidermal Langerhans cells (LCs), subcutaneous application is supposed to preferentially prime dermal interstitial dendritic cells (DCs) for further immunological T-cell mediated response.

Basing upon these data, different algorithms to evaluate patients with suspected immediate- and delayed-type LA reactions by the means of skin testing (patch and intradermal testing, prick testing) and different challenging protocols have been proposed [2,4].

We herein describe a patient with a delayed-type allergy against prilocaine, as verified by skin testing and positive subcutaneous challenge. To note, in our patient the delayed-type prilocaine allergy could only be provoked by *superficial*, but not by *deep *subcutaneous injection. This fits into the concept of compartment allergy which supposes that clinical manifestations of T-cell mediated allergic reactions may depend on the density and functional state of immune-regulatory cells in the relevant tissue microenvironment.

## Case presentation

### Medical history

A 42-year old female patient presented to our department with a progressive, disseminated eczematous skin eruption beginning at her left leg eleven days before, and successively spreading to trunk as well as to upper extremities over the course of the following days. Five days prior to onset, reticular varicosis of her left lower leg was surgically treated in local anesthesia (LA) with postoperative plaster and bandage application. Topical application of high-potency steroids and emmolients yielded rapid clearing of the itchy skin eruption.

As initially spreading allergic contact dermatitis to ingredients of band-aids was suspected, subsequent stripping surgery of the right saphenous vein in LA four weeks later was undertaken without postoperative plaster application. However, on the first postoperative day the patient developed again a similar, itching progressive eczematous dermatitis still being highly suggestive for allergic contact dermatitis.

Further preoperative medication included the desinfectant Septoderm™ (2-propanol, butandiol, lanolin-polyoxyethylene) and the local anesthetic Xylonest™ 0.5% (prilocaine, methylhydroxybenzoate). Systemic medication or further topical advice, concomitant infections, prior allergies despite drug allergy against aminopenicillins and omeprazole or relevant comorbidities were denied. Dental interventions using Ultracain™ (articaine, no preservative agent) had been formerly tolerated without symptoms.

### Investigations

Histological examination of eczematous lesions revealed a dense, subepidermal, predominantly lymphocytic inflammatory infiltrate with perivascular accentuation extending to the deep corium together with prominent spongiotic epidermal alteration, being highly suggestive for allergic contact dermatitis.

Laboratory investigations were without pathological findings, respectively.

Patch testing at the upper back with readings at days 2, 3 and 4 comprising the standard German Contact Allergy Group (DKG) series as well as key substances of plaster ingredients (acrylates, benzoylperoxide, substituted glycidylethers, diaminodiphenylmethan) and methylhydroxybenzoate were negative except sensitization against nickel and fragrances without clinical relevance.

However, patch testing with 1% prilocaine solution resulted in a crescendo pattern of 2-fold-positive test reactions at days 3 and 4, confirmed by positive intracutaneous testing demonstrating erythema and induration after 4 days. Consecutively performed patch and intradermal skin testing with other local anesthetics harbouring different chemical structures both of the amide and ester type (lidocaine 1%, mepivacaine 1%, bupivacaine 0,5%, procaine 1%, articaine 1%) remained negative during 4 days reading. For intradermal testing, a 1:10 dilutaion was used, i.e. lidocaine 0,1%, mepivacaine 0,1%, bupivacaine 0,05%, procaine 0,1%, articaine 0,1%

### Provocation

To verify clinical relevance, we challenged the patient with subcutaneous injections of Xylonest™ 1% (prilocaine) and as control the negatively tested and formerly tolerated Ultracain™ 1% (articaine) (1 ml each as one single dose at the lateral upper arm) after instruction and written informed consent.

**Figure 1:**
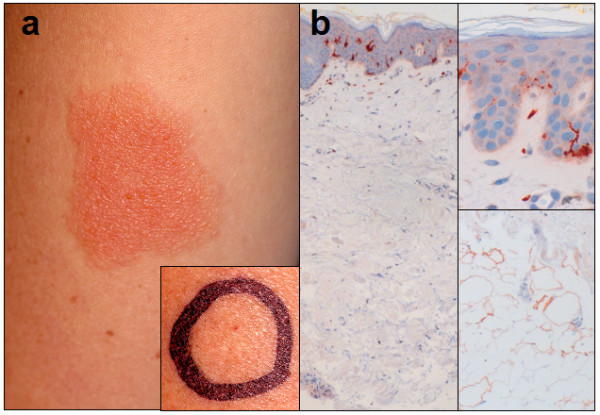
**Clinical findings in delayed-type allergy against local anesthetics and histological picture of skin compartments showing the distribution of S-100 positive dendritic cells. a **Delayed-type hypersensitivity reaction 4 days after superficial subcutaneous provocation with prilocaine (1 ml). **Inlet **Negative result after deep s.c. injection of prilocaine after 4 days. **b **Differential density of DCs in different skin compartments. Immunohistochemistry with staining of S-100 antigen. **Left **Overview of the skin layers with declining density of S-100 positive antigen-presenting cells from epidermis to subcutaneous tissue. Magnification 4 ×. **Upper right **High density of epidermal dendritic cells (Langerhans cells) in epidermis, papillary dermis and reticular dermis. Magnification 40 ×. **Lower right **In deeper subcutaneous compartments, dermal interstitial S-100 positive DCs are not detectable. Magnification 40 ×.

Incremental inflammatory reactions after 1-4 days were provoked by *superficial *subcutaneous injection of prilocaine (Figure 1a), so that diagnosis of delayed-type allergy against prilocaine without cross-reactivity against other local anesthetics of different substance groups was made. To note, provocation testing with articaine remained negative over 4 days.

To test reaction patterns of different cutaneous compartments, we also applied equal amounts (1 ml single dose) of prilocaine as *deep *subcutaneous injections. Strikingly, *deep *subcutaneous injection did not produce dermatitis at the injection side over the course of 4 days (Figure 1a, **inlet**). Neither were swelling at deeper subcutaneous layers, local hyperemia nor subjective symptoms like pruritus or burning observed.

## Conclusions

Topically applied LA may produce allergic contact dermatitis. While the ester-type LA benzocaine is a potent sensitizer, the amide-type of LA are rare sensitizers concerning either topical application or subcutaneous injection.

Depending on the application route, immunologic sensitization against antigenic LA determinants is conferred by distinct antigen presenting cells in different skin compartments, namely Langerhans cells (LCs) in the epidermal compartment and interstitial, dermal dendritic cells (DCs) in deeper subcutaneous tissue. These DC subsets are known to express different surface molecules and cytokines. Immunological studies have shown that epidermal LCs express CD1a, Langerin and E-cadherin while dermal interstitial DCs are positive for DC-sign, CD11b, factor XIIIa and CD14 [5] and differ in their expression of characteristic immune-regulatory toll like receptors (TLRs). These two kinds of DCs may play different roles in regulating humoral and cellular immunity and also tolerance [6]. Epithelial cells including keratinocytes as well as further cellular components of the innate and adaptive immune system modify the DC-mediated immunological reponse [7,8]. Furthermore, *epidermal *LC density determines their capacity to induce contact hypersensitivity as demonstrated both *in vitro *as well as in different settings in animal models [9,10]. So far it is unknown, wether *dermal *DC density has an impact on launching T-cell mediated responses. In healthy tissue, DC density gradually declines from epidermis to deeper skin layers. While epidermis and the superficial subcutaneous tissue harbour a plethora of professional and non-professional antigen-presenting cells (APCs), only scarce DCs can be detected in deeper subcutaneous compartments (Figure 1b).

Therefore, vaccination strategies concerning diverse application fields like cancer, allergy and infection use either intradermal or superficial subcutaneous injection or add nonspecific, immunostimulatory substances like imcomplete Freund adjuvant, cytokines or Toll-like receptor agonists in order to enhance cellular immune responses. In this context, immune response to various vaccines, e.g. influenza virus antigen, often significantly depends on the site of injection exhibiting a much stronger immune response when applied to the dermal in comparison to muscular tissue [11].

Maybe, low DC density in deeper skin layers might explain the absence of clinical symptoms on *deep *subcutaneous application of prilocaine in our patient, while epicutaneous contact and *superficial *subcutaneous injection successfully activated cellular response by tissue APCs in our patient. Moreover, the DC subtype in deeper cutaneous tissue may differ in functional state and contribute to immunological tolerance as known by DC-derived, IL-10-mediated induction of regulatory T-cells (Tregs) [12,13]. As another example of compartment allergy, we and others have shown that delayed-type hypersensitivity to subcutaneously injected heparin does not result in clinical allergy when heparin is administered intravenously [14,15]. Blood DC subtypes - different from skin APCs - circulating in only low frequencies may be responsible for the observed tolerance [6]. Hence, one may speculate, that our patient might not only tolerate *deep *subcutaneous injection of prilocaine (i.e. in case of tumescence anesthesia), but moreover might tolerate epidural administration of prilocaine for regional anesthesia.

In summary, our presented case report demonstrates differential immunological reactions towards an allergen depending on the anatomical compartment and thereby provides another example for the concept of compartment allergy.

## Abbreviations

(LA): Local anesthetic; (APC): antigen presenting cells; (DC): dendritic cells; (LC): Langerhans cells; (s.c.): subcutaneous;

## Consent

Written informed consent was obtained from the patient for publication of this case report and any accompanying images. A copy of the written consent is available for review by the Editor-in-Chief of this journal.

## Competing interests

The authors declare that they have no competing interests.

## Authors' contributions

MW and ZG gathered the patient's history and prepared the clinical pictures. AT supervised the interpretation of the data and the design of the allergologic work-up. MW organized and finalised the manuscript and prepared histological pictures. All authors have been involved in drafting the manuscript and revising it critically for important intellectual content. All authors read and approved the final version of the manuscript.
